# Safety in numbers: multiple occurrences of highly similar homologs among *Azotobacter vinelandii* carbohydrate metabolism proteins probably confer adaptive benefits

**DOI:** 10.1186/1471-2164-15-192

**Published:** 2014-03-14

**Authors:** Mali Mærk, Jostein Johansen, Helga Ertesvåg, Finn Drabløs, Svein Valla

**Affiliations:** Department of Biotechnology, Norwegian University of Science and Technology, NO-7491 Trondheim, Norway; Department of Cancer Research and Molecular Medicine, Norwegian University of Science and Technology, NO-7491 Trondheim, Norway

## Abstract

**Background:**

Gene duplication and horizontal gene transfer are common processes in bacterial and archaeal genomes, and are generally assumed to result in either diversification or loss of the redundant gene copies. However, a recent analysis of the genome of the soil bacterium *Azotobacter vinelandii* DJ revealed an abundance of highly similar homologs among carbohydrate metabolism genes. In many cases these multiple genes did not appear to be the result of recent duplications, or to function only as a means of stimulating expression by increasing gene dosage, as the homologs were located in varying functional genetic contexts. Based on these initial findings we here report in-depth bioinformatic analyses focusing specifically on highly similar intra-genome homologs, or synologs, among carbohydrate metabolism genes, as well as an analysis of the general occurrence of very similar synologs in prokaryotes.

**Results:**

Approximately 900 bacterial and archaeal genomes were analysed for the occurrence of synologs, both in general and among carbohydrate metabolism genes specifically. This showed that large numbers of highly similar synologs among carbohydrate metabolism genes are very rare in bacterial and archaeal genomes, and that the *A. vinelandii* DJ genome contains an unusually large amount of such synologs. The majority of these synologs were found to be non-tandemly organized and localized in varying but metabolically relevant genomic contexts. The same observation was made for other genomes harbouring high levels of such synologs. It was also shown that highly similar synologs generally constitute a very small fraction of the protein-coding genes in prokaryotic genomes. The overall synolog fraction of the *A. vinelandii* DJ genome was well above the data set average, but not nearly as remarkable as the levels observed when only carbohydrate metabolism synologs were considered.

**Conclusions:**

Large numbers of highly similar synologs are rare in bacterial and archaeal genomes, both in general and among carbohydrate metabolism genes. However, *A. vinelandii* and several other soil bacteria harbour large numbers of highly similar carbohydrate metabolism synologs which seem not to result from recent duplication or transfer events. These genes may confer adaptive benefits with respect to certain lifestyles and environmental factors, most likely due to increased regulatory flexibility and/or increased gene dosage.

**Electronic supplementary material:**

The online version of this article (doi:10.1186/1471-2164-15-192) contains supplementary material, which is available to authorized users.

## Background

Genes sharing a common origin, without any further specification of their evolutionary relationship, are classified as homologs, while paralogs and orthologs constitute subcategories of homologs. Orthologs evolve from a common ancestral gene via vertical descent (speciation), while paralogs evolve by duplication events taking place after speciation [1]. Apparent gene duplications may also result from horizontal gene transfer (lateral transfer of genetic material between species), and such homologs are classified as xenologs.

Gene duplication was first developed as a coherent concept by Ohno more than 40 years ago [2], but the prevalence and importance of gene duplication was not clearly demonstrated until fully sequenced genomes became available. Estimates of numbers of duplicated genes in genomes of bacteria, archaea and eukarya have shown that large proportions of genes have been generated by gene duplication in all three domains [3]. In eubacterial systems at least three, often partly overlapping, biologically relevant roles of gene duplication can be distinguished: (a) to confer a solution to a selective problem, (b) to facilitate further genetic adaptation, and (c) to give rise to genetic innovation and novel biochemical function [4].

Following a duplication event there are three possible fates for the duplicate genes: deletion, silencing (nonfunctionalization), or selection [2]. In cases of selection three major outcomes have been described: evolution of a new beneficial function in one duplicate (neofunctionalization), partitioning of ancestral functions between the duplicates (subfunctionalization), or conservation of ancestral function(s) in both duplicates (gene conservation) [2, 5–7]. It is however likely that paralog evolution may involve both subfunctionalization and neofunctionalization, either concurrently or sequentially [1]. There is also evidence for a mechanism where there is no “invention” of new gene function(s) or direct partitioning of ancestral functions, but rather amplification followed by improvement of extant weak or secondary functions in duplicate genes [8–10]. Most studies on the outcome of retained gene duplications have been carried out in eukaryotic systems, where gene and genome duplications are currently regarded as the major sources for development of new functions. However, mechanisms for gene duplication in plants and animals and references therein] differ from those in prokaryotes [11, 12] and references therein. In addition, polyploidy and cellular differentiation plays a major role in paralog evolution in many eukaryotes.

Gene duplications are among the most common mutations in bacteria, but show high intrinsic instability. Most tandem duplication events in bacterial genomes are associated with naturally occurring repetitive sequences, such as rRNA genes, transposable elements and other repetitive elements, but gene duplication can also occur by processes capable of random end-joining in the complete absence of any repetitive sequence [4]. Duplicated genes in bacteria appear to be created mainly by small-scale gene duplication events, and the majority of retained duplicated genes occur as single genes, not duplicated operons [13]. The total number of paralogs in microbial genomes has been shown to correlate with genome size [8].

Microbial genomes also acquire intra-genome homologs via horizontal gene transfer, leading to xenologs. For most bacterial and archaeal genomes there appears to be a high level of horizontal gene transfer events in general [14, 15]. Xenologs can also be described as pseudoparalogs. It will usually not be possible to discern between pseudoparalogs and true paralogs in a single-genome analysis [1]. Thus, Lerat *et al*. [15] have proposed the term “synologs” to describe intra-genome homologs arising from either duplication or horizontal transfer. The term was originally introduced by Gogarten [16] with a different definition, but the definition by Lerat *et al*. seems to be preferred and will be used in this study. For γ-proteobacteria (the class *Azotobacter vinelandii* [see below] belongs to) it has been shown that horizontally acquired genes only rarely have pre-existing homologs within the recipient genome. But because synologs are rare in general, lateral gene transfer still contributes substantially to synology [15]. Furthermore, subsequent duplications appear to be more common among laterally transferred genes. This is probably due to selection for gene dosages, as recently imported genes are likely to have poorly optimized functions [17].

During annotation work on the genome of the metabolically versatile nitrogen-fixing soil bacterium *A. vinelandii* DJ [18], we observed that the genome of this organism appeared to have a high frequency of highly similar synologs among proteins involved in carbohydrate metabolism. The 5.4 Mb genome contains approximately 5000 protein-coding genes, out of which ~70% have been given a functional assignment [18]. To further investigate the abovementioned observation 943 bacterial and archaeal genomes that were available in the SEED database [19] at the time of the study were analysed for the occurrence of carbohydrate metabolism synologs. The general opinion is that in most cases, duplicated genes will diverge over time. This is consistent with Hooper and Berg’s observation that bacterial and archaeal genomes show roughly the same pattern of paralogs in groups of decreasing amino acid identity [8]. Thus, a high incidence of highly similar synologs could be explained either by a recent burst of duplications or repeated horizontal gene transfers, or by a mechanism for gene conservation operating on these specific genes. The latter would imply that retention of highly similar synologs benefits the organism adaptively. The results of this study support that sequence conservation of multiple gene copies can be an adaptive strategy for bacteria to enhance their ability to cope with various environmental conditions.

## Results and discussion

### The *A. vinelandii*DJ genome contains abundant carbohydrate metabolism synologs, many of which display a high degree of sequence identity

**Figure 1:**
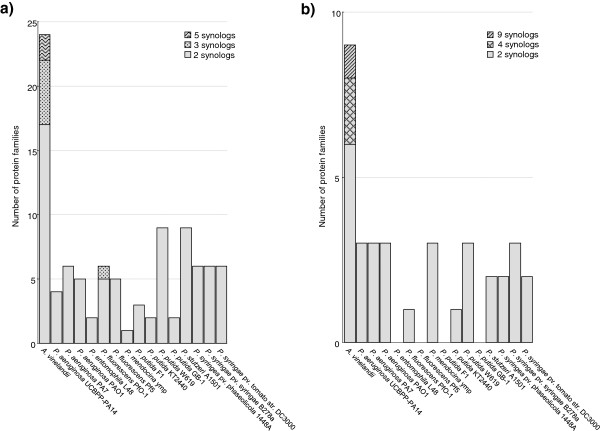
**Distribution of carbohydrate metabolism and transport protein families in** ***A. vinelandii*** **and pseudomonad genomes.** The number of intra-genome protein families (identified using OrthoMCL) assigned to the functional categories **a)** carbohydrate metabolism and **b)** carbohydrate transport in the genomes of *A. vinelandii* DJ and 15 fully sequenced strains in the genus *Pseudomonas*. This illustrates that for carbohydrate metabolism the number of synologs is clearly higher in the *A. vinelandii* genome compared to the *Pseudomonas* strains included in this study. The total number of families for each genome is shown as stacked columns, with block patterning indicating the number of proteins in the identified families. Members of the same intra-genome protein family were regarded as synologs.

Annotation work on the genome of soil bacterium *A. vinelandii* DJ [18] revealed a high frequency of synologs among proteins involved in carbohydrate metabolism. To determine whether this is also the case for the closely related pseudomonads, and whether synologs are common for other functional categories in these genomes, the occurrence of protein families in the genomes of *A. vinelandii* DJ and 15 fully sequenced strains in the genus *Pseudomonas* was investigated using OrthoMCL [20], which uses pairwise sequence alignments to identify groups of similar proteins (protein families) in proteomes. In this analysis, members of the same intra-genome protein family were regarded as synologs. The number of identified families was markedly higher in *A. vinelandii* DJ than in the pseudomonad strains with regard to proteins involved in both carbohydrate metabolism (Figure 1a) and carbohydrate transport (Figure 1b), but not for any of the other functional categories. In addition, carbohydrate related protein families with more than two members were more common in *A. vinelandii* (Figure 1). Another interesting outcome of this initial study was that more than half of the identified *A. vinelandii* protein families assigned to carbohydrate metabolism contained synologs that shared ≥90% protein sequence identity.

### An abundance of carbohydrate metabolism synologs is uncommon in prokaryotic genomes

To investigate the occurrence of synologs among carbohydrate metabolism genes in a wider range of organisms, data was extracted from the SEED database [19]. One of the challenges in comparative genomics is that the extent and quality of annotation, as well as the approaches, can vary widely between genomes. The SEED provides an environment in which all genomes have been subjected to the same annotation pipeline, with curation executed across many genomes. Extracting data from this database can thus be expected to yield comparable gene sets for the included genomes. Sequences belonging to the “Carbohydrates” category (including genes involved in transport) were extracted for the 57 archaeal and 886 bacterial genomes available in the SEED database at the time of the study.

The SEED database sorts protein-coding sequences into FIGfams where all members are believed to have the same functional role(s). Sequences from the same genome belonging to the same FIGfam, regardless of level of sequence identity, were regarded as synologs. Out of the 943 genomes, 46 (i.e. 4.9%) were found not to contain any synologs among carbohydrate metabolism genes. The gene sets extracted from SEED included both functional genes and genes annotated as possible pseudogenes, but the gene sequences were translated before the data set was analysed. This ensured that pseudogene sequences containing frameshifts or deletions resulting in severely changed proteins would not be included in the identified sets of highly similar synologs, as the sequence set was also checked for severely truncated proteins (see Methods). Analysing the data set with emphasis on total number of synologs and number of synolog groups (sets of two or more intra-genome synologs) yielded practically indistinguishable qualitative results due to the strong positive correlation between these values (R^2^ = 0.95 for a linear regression).

When all synologs were included (regardless of level of sequence identity), *A. vinelandii* DJ was found to contain 145 carbohydrate metabolism synologs distributed into 52 synolog groups. These synologs constituted 45.6% of the total carbohydrate metabolism genes in the genome, hereafter referred to as the synolog fraction. This is significantly higher than the median numbers found when analysing all 943 genomes (Table 1), and placed *A. vinelandii* DJ in the upper 9% when strains were ranked based on number of synolog groups.

**Table 1:** **Summary of statistical data for SEED data sets** ^**1**^

	All synolog pairs			≥90% protein sequence identity between synolog pairs		
	Median ± MAD	Min	Max	Median ± MAD	Min	Max
Number of carbohydrate metabolism synologs	49.0 ± 35.0	0	394	0.0 ± 0.0	0	47
Number of carbohydrate metabolism synolog groups	20.0 ± 13.0	0	128	0.0 ± 0.0	0	16
Average protein sequence identity between synolog pairs^2^ [%]	36.8 ± 4.2	13.4	100.0	97.3 ± 1.8	90.0	100.0
Synolog fraction of carbohydrate metabolism genes [%]	30.0 ± 9.7	0.0	85.7	0.0 ± 0.0	0.0	34.6

^1^Median, minimum and maximum values for the carbohydrate metabolism gene set extracted from 943 prokaryote genomes in the SEED database [19], with no set cutoff and with a cutoff set at 90% protein sequence identity between synologs. Synologs are here defined as intra-genome sequences assigned to the same FIGfam (see text). The synolog fraction describes the ratio of the total number of synologs relative to the total number of genes in a genome. MAD is median absolute deviation. The median number of carbohydrate metabolism genes in the data set was 160.0 ± 74.0. The minimum and maximum numbers of carbohydrate metabolism genes observed among the included genomes were 4 and 585 genes, respectively.

^2^Calculated from the genomes containing carbohydrate metabolism synologs at the given cutoff.

**Figure 2:**
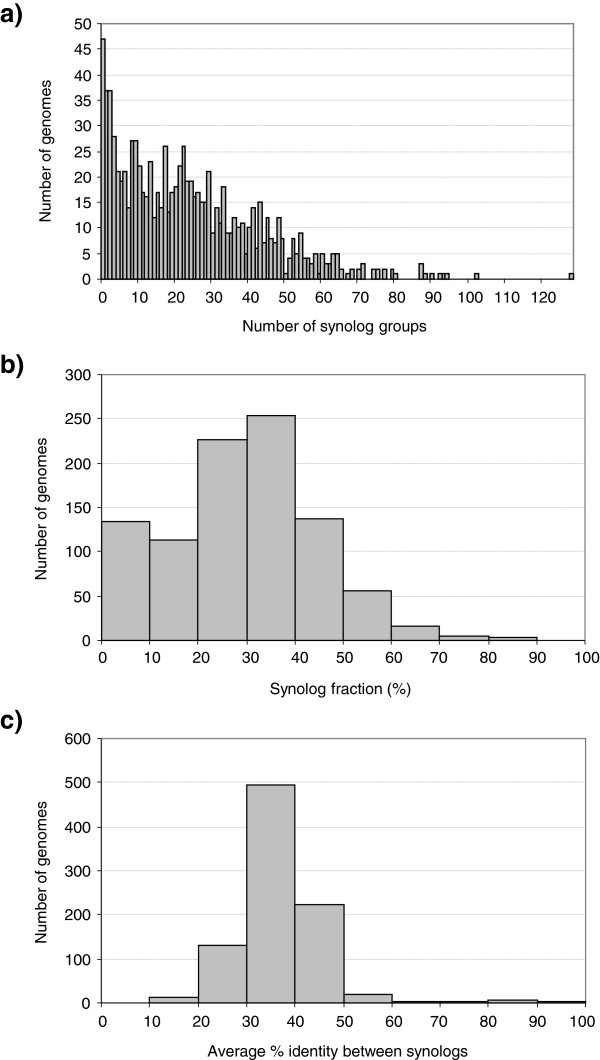
**Distribution of synolog groups**, **synolog fractions and average synolog sequence identity for carbohydrate metabolism genes.** Distribution of **a)** number of synolog groups, **b)** synolog fractions and **c)** average synolog sequence identity at the protein level for carbohydrate metabolism synologs in a data set consisting of 943 [**a)-b)** ] or 897 [**c)** ] bacterial and archaeal genomes, illustrating that a high fraction of very similar carbohydrate metabolism synologs is rare among the genomes included in this analysis. Synologs are here defined as intra-genome sequences assigned to the same FIGfam in the SEED database [19]. The synolog fraction describes the ratio of the total number of carbohydrate metabolism synologs relative to the total number of carbohydrate metabolism genes in a genome.

Different strains of the same species generally displayed similar synolog frequencies and levels of average synolog identity. Only 13% of the genomes contained more than 46 (median + 2× MAD) synolog groups (Figure 2a) and synologs constituted more than 49.4% (median + 2× MAD) of carbohydrate metabolism genes in only 9% of the genomes (Figure 2b).

Additionally, the results indicated that carbohydrate metabolism synologs with a high level of sequence identity is not a common phenomenon in bacterial and archaeal genomes, as the average identity between synolog pairs was ≤50% in 96% of the 897 genomes which contained such synologs (Figure 2c). Furthermore, 24 out of the 35 genomes with average sequence identity >50% contained less than ten synolog groups.

### Large numbers of carbohydrate metabolism synologs with high identities are rare in prokaryotic genomes, and is not correlated with total number of protein coding genes

**Figure 3:**
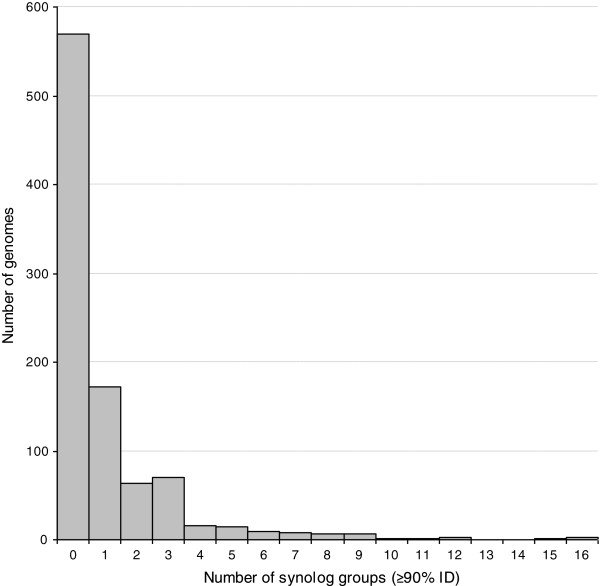
**Occurrence of synologs with** ≥**90** **%** **identity among carbohydrate metabolism proteins in bacteria and archaea.** Number of carbohydrate metabolism synolog groups with internal protein sequence identity ≥90% identified in 943 investigated prokaryotic genomes. Synolog groups are here defined as two or more intra-genome sequences assigned to the same FIGfam in the SEED database [19].

Based on the results from the initial investigations described above, the data set extracted from SEED was filtered to identify synolog groups with protein sequence identities ≥90%. Such synologs were found to be present in 374 (i.e. 40%) of the genomes. It should be noted that the average sequence identity between synolog pairs in this data set was ~97% (Table 1), which means that the majority of the synologs has a considerably higher degree of identity than the applied cutoff value. The high correlation between number of synologs and number of synolog groups was still present at this cutoff (R^2^ = 0.97 for a linear regression). As can be seen in Table 1, applying this cutoff reduced the median number of synolog groups from 20 ± 13 to 0 ± 0, demonstrating that high frequencies of very similar carbohydrate metabolism synologs are indeed rare. This is also evident from Figure 3, which shows how the number of highly similar synolog groups for carbohydrate metabolism is distributed across genomes.

As could be expected, there was a positive correlation between the number of carbohydrate metabolism synolog groups in the genomes and the total number of carbohydrate metabolism genes, total number of protein-coding genes or genome size (R^2^ = 0.80, 0.56 and 0.51 respectively for a linear regression). There was however no clear correlation when only synologs displaying protein sequence identities ≥90% were included (R^2^ = 0.14, 0.15 and 0.13 respectively), so a high frequency of highly similar synologs in a genome cannot simply be attributed to a large genome or a large number of genes in general.

### The *A. vinelandii*DJ genome harbours an unusually large number of highly similar carbohydrate metabolism synologs

When the threshold of minimum 90% protein sequence identity between synologs was applied, the *A. vinelandii* DJ genome was found to harbour 38 carbohydrate metabolism synologs distributed into 15 synolog groups (Table 2). At this sequence identity cutoff, 10 or more synolog groups were observed in only 1% of the analysed genomes (Figure 3). Thus, the results confirm that the *A. vinelandii* DJ genome does indeed harbour an unusually large number of highly similar carbohydrate metabolism synologs compared to the majority of sequenced bacterial and archaeal genomes. This observation also holds true if the analysed strains are ranked according to synolog fractions rather than number of synolog groups.

**Table 2:** **The genomes with the highest levels of very similar carbohydrate metabolism synologs** ^**1**^

Genome	Number of synolog groups											
	Total	Central carbohydrate metabolism	Organic acids	Di- and oligo-saccharides	Fermentation	One-carbon metabolism	CO _2_ fixation	Amino-sugars	Poly-saccharides	Carbohydrates - no sub-category	Sugar alcohols	Mono-saccharides
***Soil*** **/** ***sediments***												
*Clostridium beijerinckii* NCIMB 8052	16	3	-	2	5	-	-	-	-	-	-	6
*Azotobacter vinelandii* DJ	15	8	-	2	5	-	-	-	-	-	-	-
*Burkholderia xenovorans* LB400	12	3	1	-	4	-	-	-	-	-	1	3
*Bacillus cereus* E33L^2^	10	2	-	-	-	-	-	-	-	-	7	1
*Nakamurella multipartita* DSM 44233	9	4	4	-	-	-	-	-	-	-	1	-
*Rhodoferax ferrireducens* DSM 15236	9	3	-	-	3	-	-	-	-	-	-	3
*Burkholderia cepacia* R18194^2^	9	-	-	-	5	1	-	-	-	-	-	3
*Nitrobacter hamburgensis* X14	9	2	-	-	-	-	6	-	-	-	-	1
*Frankia* sp. EAN1pec	9	8	-	-	1	-	-	-	-	-	-	-
*Paracoccus denitrificans* PD1222	8	4	1	-	-	3	-	-	-	-	-	-
*Ralstonia eutropha* JMP134	8	3	1	-	2	2	-	-	-	-	-	-
*Burkholderia vietnamiensis* strain G4^2^	8	2	-	-	4	1	-	-	-	-	-	1
***Marine*** **/** ***aquatic***												
*Shewanella baltica* OS155	12	10	-	2	-	-	-	-	-	-	-	-
*Methylobacillus flagellatus* KT	8	7	-	1	-	-	-	-	-	-	-	-
***Pathogens***												
*Vibrio cholerae* MZO-3^3^	16	6	-	1	1	1	-	-	-	-	-	7
*Shigella dysenteriae* M131649	16	2	-	5	-	1	-	-	-	1	2	5
*Escherichia coli* B7A^3^	12	3	-	2	-	-	-	-	-	-	2	5
*Streptococcus pneumoniae* OXC141^3^	11	3	-	6	1	-	-	1	-	-	-	-
*Escherichia coli* E110019^3^	8	-	-	-	-	-	-	-	-	-	8	-
***Commensals***												
*Streptococcus mitis* NCTC 12261^3^	9	1	-	6	-	-	-	-	-	1	1	-

^1^The table lists the twenty genomes with the largest number of synolog groups among carbohydrate metabolism genes when a threshold of at least 90% amino acid sequence identity was used. The data set was extracted from the SEED database [19] and synologs were defined as intra-genome sequences assigned to the same FIGfam (see text). The total number of such synolog groups in these genomes as well as their distribution in the eleven subcategories defined in the SEED database is shown. The median number of synolog groups for the genomes in this data set was 2.0 ± 1.0.

^2^Opportunistic pathogen.

^3^All synologs in this table were evaluated manually with regards to genomic context. The manual evaluation revealed that several of the synologs in *V. cholerae* MZO-3, *E. coli* B7A, *S. pneumoniae* OXC141, *S. mitis* NCTC 12261 and *E. coli* E110019 might be mistakenly identified as highly similar synologs due to overlapping contigs or the presence of truncated sequences. These sequences were therefore disregarded in interpretation of the results.

### The majority of highly similar carbohydrate metabolism synologs in *A. vinelandii*DJ belong to core metabolic processes

SEED divides the “Carbohydrates” category into eleven subcategories, which enabled us to investigate the distribution of synologs in various parts of the carbohydrate metabolism. In the *A. vinelandii* DJ genome, all but two synolog groups occurred in the subcategories “Central carbohydrate metabolism” and “Fermentation” when the cutoff of 90% sequence identity was applied. In the “Central carbohydrate metabolism” subcategory, genes for glucose 6-phosphate dehydrogenase (*zwf*), transketolase (*tktA*), glucose 6-phosphate isomerase (*pgi*), triosephosphate isomerase (*tpiA*), 2,3-bisphosphoglycerate-independent phosphoglycerate mutase (*pgm*), enolase (*eno*), pyruvate kinase (*pykA*) and transaldolase (*talB*) were found to have synologs with minimum 90% sequence identity, while genes encoding electron transfer flavoprotein subunits (*etfA* and *etfB*), electron transfer flavoprotein ubiquinone oxidoreductase, acetaldehyde dehydrogenase (*mhpF*) and 4-hydroxy-2-oxovalerate aldolase (*xylK*/*mphE*) were found to have such synologs in the “Fermentation” subcategory.

**Figure 4:**
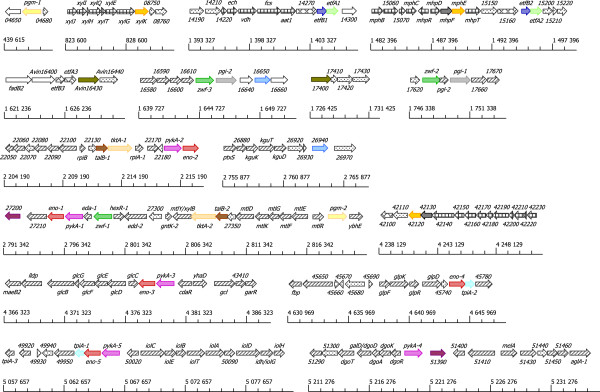
**Genomic contexts for carbohydrate metabolism synologs in** ***A. vinelandii*** **DJ with ≥** **90** **%** **identity.** The figure shows that the highly similar *A. vinelandii* DJ carbohydrate metabolism synologs identified in this study are found in differing genomic contexts. Synologs are here defined as intra-genome sequences assigned to the same FIGfam in the SEED database [19] and a threshold was set to include only synologs displaying at least 90% protein sequence identity. Synolog groups are highlighted as same-coloured arrows. Striped arrows represent other genes annotated as carbohydrate metabolism genes, checkered arrows represent genes annotated as aromatic compounds metabolism genes, meshed arrows represent genes annotated as electron transport genes, white arrows represent genes annotated as belonging to other functional categories, and dotted arrows represent genes annotated as encoding hypothetical proteins or proteins of unknown function. Genes that had not been assigned a gene name in the annotated *A. vinelandii* DJ genome are marked with the number corresponding to their respective geneIDs, which in the genome annotation are written on the form Avin*#####*[18].

For both subcategories the genes in a given synolog group were found to be localized in partly or completely different genomic contexts, and often as parts of differing probable operons. In several cases, genes from two or more synolog groups were localized in the same gene cluster or putative operon (Figure 4). The majority of the “Fermentation” subcategory synologs were clustered with genes involved in the utilization of aromatic compounds as energy sources, while the “Central carbohydrate metabolism” synologs were always clustered with other carbohydrate metabolism genes; mainly genes involved in transport and degradation of various sugars. The varying, but metabolically relevant genomic contexts indicate that these highly similar synologs are not the result of very recent duplications and/or repeated horizontal gene transfers, as adaptive selection resulting in favourable genomic arrangements appears to have taken place along with conservation of the duplicated sequences, even though the former can be assumed to be a very slow process [21, 22].

It has been suggested that organizing genes in operons could be inherently suboptimal, as it may prevent fine-tuning of expression, and that operons might exist despite this disadvantage because they facilitate the evolution of co-regulation [23, 24]. Thus, it is also possible that retaining highly similar synologs in different operonic contexts might be advantageous with regard to co-regulation while at the same time leaving more room for fine-tuning production of a particular protein. The localization of highly similar synologs in varying but metabolically relevant genomic contexts was also observed to be common among other genomes containing large numbers of such synologs.

### Comparison to other *A. vinelandii*genomes supports conservation of sequences

At the time of this study, *A. vinelandii* DJ was the only published genome from the genus *Azotobacter*, making it difficult to assess whether the occurrence and genomic localization of highly similar synologs is similar in other strains, which would support conservation as an important factor. However, during the preparation of this paper the genome sequence of *A. vinelandii* CA (also known as UW or OP) was published [25]. All the genome fragments depicted in Figure 4 were found to have 100% identical homologous sequences in strain CA. This is not surprising, as *A. vinelandii* DJ is a variant of strain CA generated in 1984 [18]. Another strain, *A. vinelandii* E, was shotgun sequenced as part of the *A. vinelandii* genome project resulting in the publication of the *A. vinelandii* DJ genome. Strain CA, and thus DJ, are laboratory strains derived from *A. vinelandii* O [26] of which the earliest report dates back to 1952 [27], when it was part of the bacterial collection at the University of Wisconsin, Madison, US. Strain E was isolated from a soil sample collected in Trondheim, Norway in the 1960’s.

Due to our participation in the *A. vinelandii* genome project we have access to the strain E shotgun sequence, which currently consists of 3947 contigs with lengths ranging from 100 bp to 57 378 bp. Analysis of the available contigs showed that several of the above mentioned synologs are found in the same arrangements in this organism, and with gene sequences nearly identical to those in *A. vinelandii* DJ. This also supports that these highly similar *A. vinelandii* carbohydrate metabolism synologs did not originate from recent duplication/horizontal transfer events, as it is reasonable to assume that a considerable number of generations have passed since these two strains’ last common ancestral cell. There is not much data available on how quickly, and by what mechanisms, free-living non-pathogenic bacteria evolve in their natural environments, but a recent study by Denef and Banfield [28] showed that a series of important divergence events occurred over a time scale of years to decades in *Leptospirillum* biofilms in a mine drainage system. These included nucleotide substitutions, recombination and insertion/deletion events.

We considered the possibility that the sequences of the abovementioned conserved proteins are so crucial for function that even minor changes would be deleterious and thus heavily selected against. However, a comparison of Zwf, TktA and PykA sequences from *A. vinelandii*, *P. stutzeri*, *P. aeruginosa*, *P. fluorescens* and *E. coli* revealed sequence identities as low as 40%, indicating considerable room for changes without loss of functionality.

### The occurrence and distribution of highly similar carbohydrate metabolism synologs might be connected to lifestyle and environmental factors

*A. vinelandii* is widely distributed in soil and water and is a very metabolically versatile species. The majority of the other bacteria with large numbers of highly similar carbohydrate synologs (Table 2) are also found in soil, sediments or water, but this could be due to a prevalence of this kind of organisms among sequenced bacterial genomes. Gene duplication and divergence are however assumed to be important for adaptation in environments of scarce, but diverse nutrients, such as soil [29]. Even though the synologs studied here are highly similar it is possible that such carbohydrate metabolism synologs could confer advantages with regard to utilization of the available carbon sources, as independently regulated synologs might allow a higher degree of flexibility and efficiency in situations of simultaneous utilization of varying combinations of nutrients. As discussed above, the identified *A. vinelandii* DJ synologs were often found in varying, but metabolically relevant genomic contexts. The same was observed for the other soil bacteria in Table 2. Divergent expression of duplicate genes is well-known in eukaryotes, and expression divergence has been shown to be common even for highly similar gene pairs in yeast [30]. Duplications have also been shown to have a key role in evolution of gene regulatory networks in both *E. coli* and yeast [31]. In addition to regulatory advantages, the organisms could also gain benefits simply from increased gene dosages.

Table 2 lists the twenty genomes with the highest number of synolog groups among carbohydrate metabolism genes when the threshold of minimum 90% sequence identity was applied, and shows the distribution of these synologs in the SEED database’s eleven “Carbohydrates” subcategories. For most of the genomes in Table 2 the majority of the observed synolog groups are clustered in one or two of the subcategories, although which subcategory/-ies varies for the different strains (discussed below). This could indicate that in some organisms, retention of highly similar carbohydrate metabolism synologs is not a completely random process, but rather that certain genes are more prone to be retained and conserved than others due to selective pressure or other mechanisms.

As can be seen in Table 2, highly similar carbohydrate metabolism synologs in bacterial genomes are by far most common in the subcategory “Central carbohydrate metabolism”, as already observed for *A. vinelandii*. This could reflect adaptive benefits of increased gene dosage or regulatory flexibility in these central processes, although it could also be attributed to the fact that the central metabolism has been studied more extensively than peripheral processes, making it more likely that genes belonging to this subcategory will be well annotated in the genomes.

However, clear links to environment and lifestyle could be found in some cases. It was observed that the majority of the highly similar synologs identified in the genome of *Nitrobacter hamburgensis* X14, a bacterium which can oxidize nitrite while fixing carbon dioxide (CO_2_) as its sole carbon source [32], occur in the subcategory “CO_2_ fixation”. This is an important metabolic process in this species, as exposure to CO_2_ (or addition of sodium carbonate) has been shown to be required for optimum growth of the organism even in the presence of organic carbon [33]. Furthermore, the majority of highly similar synologs in the *Bacillus cereus* E33L genome were found among genes encoding myo-inositol catabolism proteins. *B. cereus* is found in soil, where myo-inositol is abundant, and one of the virulence factors of *B. cereus* E33L is a phosphatidyl-inositol-specific phospholipase [34].

All the genomes in Table 2 belong to bacteria, but similar observations were also made for archaeal genomes, where the largest numbers of highly similar carbohydrate metabolism synologs were found in the genomes of *Methanocella* sp. RC-I (7 synolog groups), *Methanosarcina acetivorans* C2A (6 synolog groups), *Methanococcoides burtonii* DSM 6242 (5 synolog groups) and *Methanosarcina mazei* Go1 (5 synolog groups). These four organisms use methanogenesis (a form of anaerobic respiration) as their energy-yielding metabolic process [35–38], and all the identified synologs were found among methanogenesis genes. Thus, there does appear to be a connection between environmental factors or certain aspects of a prokaryotic organism’s lifestyle and the types of genes which have highly similar synologs. This observation is also supported by earlier studies showing that retained paralogs can be beneficial for coping with environmental fluctuations [39] and that retained duplications are more common among genes involved in adaptation to environmental conditions [40].

Presumably, at least some of the identified synologs in this analysis are the results of recent duplication and/or repeated horizontal transfer events. This seems to be the case in for example the *Shigella dysenteriae* M131649 genome, where the identified synologs were observed to be localized in a wider range of genomic contexts, and nearly always non-operonic and in close proximity to mobile genetic elements.

### Highly similar synologs are generally rare in bacterial and archaeal proteomes

Finally we wanted to get a broader perspective on the occurrence of highly similar synologs overall, not just among carbohydrate metabolism genes or limited to SEED annotation. Therefore 897 bacterial and archaeal genomes available in the NCBI database at the time of the study were analysed for all intra-genome protein coding sequences (CDSs) showing at least 90% protein sequence identity along ≥90% of the full length of the compared sequences, using a strategy similar to the one employed by OrthoMCL. A set of two or more such homologs was termed a synolog group. More commonly a cutoff of 30% identity is used to identify homologous protein sequences [41, 42], but this study aimed to identify only sequences with a very high level of identity.

While the total number of protein-coding sequences in the analysed genomes has an approximately symmetric distribution [see Additional file 1: Figure S1a], the distributions of total number of synolog groups as well as synolog fractions have large positive skews (Figure S1b and c [see Additional file 1]), i.e. the majority of the genomes display very low values. Thus, the analyses showed that highly similar synologs generally comprise a very small fraction of the predicted proteomes of bacteria and archaea (hereafter referred to as the synolog fraction).

With the cutoff at 90% identity, the median synolog fraction (± median absolute deviation; MAD) was 1.9 ± 1.2%. Only 40 out of the 897 analysed genomes had synolog fractions ≥10% at a cutoff of 90% identity. The synolog fractions of these 40 genomes are presented in [see Additional file 1: Table S1]. An interesting feature of the 40 strains listed in [see Additional file 1: Table S1] is that 78% are pathogenic bacteria. Thus, even taking into account that approximately one third of all sequenced genomes belong to bacteria that live in association with eukaryotes [43], there appears to be a bias towards this kind of organisms among prokaryotic genomes with a generally high frequency of highly similar synologs. There is however otherwise great variation between these 40 strains, as they encompass both plant and animal pathogens, and intracellular as well as extracellular lifestyles. The majority of the genomes in [see Additional file 1: Table S1] have previously been reported to be rich in mobile genetic elements and/or DNA repeats [44–70], indicating a considerable contribution from such elements to very high levels of highly similar synologs overall, as could be expected.

The *A. vinelandii* DJ genome was found to have a synolog fraction of 4% at the given cutoff. While this does place *A. vinelandii* DJ in the top 20% when the analysed genomes are ranked according to synolog fraction, it is far from as extreme as the frequency observed when only carbohydrate metabolism genes were considered, showing that the exceptional abundance of carbohydrate metabolism synologs observed in this organism is not simply due to an unusually high level of highly similar synologs in general.

## Conclusions

The soil bacterium *A. vinelandii* harbours an unusually large number of highly similar carbohydrate metabolism synologs (but not highly similar synologs in general) relative to other sequenced bacterial and archaeal strains. The majority of these synologs occur in core metabolic processes. Highly similar carbohydrate metabolism synologs are generally rare in the genomes of prokaryotes, but are observed in some additional cases and there seems to be a connection between lifestyle or environmental factors and the distribution of such synologs. The majority of genomes with high levels of such synologs were from soil bacteria. In these genomes, many of the highly similar carbohydrate metabolism synologs were observed to be non-tandemly organized and localized in varying but functionally relevant genomic contexts, indicating that these synologs are not the result of recent duplications or repeated transfer events despite having nearly identical protein sequences. This supports the hypothesis that there are adaptive benefits in conservation of these highly similar synologs, most likely due to a more flexible regulation of expression and/or increased gene dosages, although this is just one of several possible strategies for adaptation. Most likely, the initial “choice” between alternative strategies for a given situation is affected by the available resources (e.g. environmental nutrients, potential for horizontal genetic exchange), the cost (increased metabolic load, slower growth rate etc.) and the benefits (e.g. enhanced nutrient utilization, more effective transition between niches) of a given strategy for the organism.

## Methods

### *A. vinelandii*and *Pseudomonas*protein families

Protein families containing two or more members were identified in the genomes of *A. vinelandii* DJ [GenBank:NC_012560], *P. aeruginosa* UCBPP-PA14 [GenBank:NC_008463], *P. aeruginosa* PA7 [GenBank:NC_009656], *P. aeruginosa* PAO1 [GenBank:NC_002516], *P. entomophila* L48 [GenBank:NC_008027], *P. fluorescens* PfO-1 [GenBank:NC_007492], *P. fluorescens* Pf5 [GenBank:NC_004129], *P. mendocina* ymp [GenBank:NC_009439], *P. putida* F1 [GenBank:NC_009512], *P. putida* KT2440 [GenBank:NC_002947], *P. putida* W619 [GenBank:NC_010501], *P. putida* GB-1 [GenBank:NC_010322], *P. stutzeri* A1501 [GenBank:NC_009434], *P. syringae* pv. phaseolicola 1448a [GenBank:NC_005773], *P. syringae* pv. syringae B728a [GenBank:NC_007005] and *P. syringae* pv. tomato str. DC3000 [GenBank:NC_004578] using OrthoMCL. OrthoMCL is a genome-scale algorithm for grouping orthologous protein sequences, which can also be used to provide groups representing species-specific gene expansion families [20]. The identified protein families were grouped into functional categories manually based on the available annotation information for the genes in each protein family.

### SEED data sets

Data from 943 archaeal and bacterial genomes were extracted from the SEED subsystem database [19] in October 2010. Synologs were defined as intra-genome sequences assigned to the same FIGfam. The Networks-Based SEED API [71] was downloaded and used to extract synolog nucleotide sequences contributing to the subsystems. The sequences were translated and ClustalW [72] was run on all intra-genome synolog pairs. Protdist from the PHYLIP package [73] was then run on each synolog pair to calculate distances (i.e. sequence identity) using the similarity table option. This approach is more sensitive than the Blast approach described below, but also much slower. However, as we here compared only a relatively small set of sequence pairs, and not all vs. all as in the NCBI data set analysis, the lack of speed was not a limiting factor. Scripting and statistical analysis was carried out with local tools written in Python [74]. This approach did not include a length requirement for the sequence alignments, but since large length differences were not prevalent among the compared sequence pairs ( [see Additional file 1: Figure S2]) this was not problematic for our analysis.

### NCBI data sets

897 complete prokaryotic proteomes were downloaded from the NCBI ftp-server [75] in June 2009. For each proteome, a blastp [76] search against itself was performed and synologs were identified using 90% sequence identity thresholds along ≥90% of the full length of the compared sequences at the protein level. This is similar to the approach used by OrthoMCL, but with stricter thresholds. At high identity thresholds, the difference between using similarity or identity is small. Maximum number of BLAST hits was set to 1000, unless the E-score from BLAST exceeded a threshold of 10^-5^.

## Availability of supporting data

The data supporting the results of this article are included within the article and its additional file.

## Electronic supplementary material

Additional file 1: Table S1: Genomes where highly similar synologs constitute ≥10% of the total number of protein-coding genes. **Figure S1.** Distribution of number of proteins, synologs and synolog groups, and synolog fractions, independent of functional categories. **Figure S2.** Distribution of difference between best and worst match for comparisons of synolog pairs retrieved from the SEED database. (PDF 173 KB)

## References

[1] Koonin EV (2005). Orthologs, paralogs, and evolutionary genomics. Annu Rev Genet.

[2] Ohno S (1970). Evolution by gene duplication.

[3] Zhang J (2003). Evolution by gene duplication: an update. Trends Ecol Evol.

[4] Andersson DI, Hughes D (2009). Gene amplification and adaptive evolution in bacteria. Annu Rev Genet.

[5] Force A, Lynch M, Pickett FB, Amores A, Yan YL, Postlethwait J (1999). Preservation of duplicate genes by complementary, degenerative mutations. Genetics.

[6] Hahn MW (2009). Distinguishing among evolutionary models for the maintenance of gene duplicates. J Hered.

[7] Lynch M, Force A (2000). The probability of duplicate gene preservation by subfunctionalization. Genetics.

[8] Hooper SD, Berg OG (2003). On the nature of gene innovation: duplication patterns in microbial genomes. Mol Biol Evol.

[9] Bergthorsson U, Andersson DI, Roth JR (2007). Ohno's dilemma: evolution of new genes under continuous selection. Proc Natl Acad Sci U S A.

[10] Näsvall J, Sun L, Roth JR, Andersson DI (2012). Real-time evolution of new genes by innovation, amplification, and divergence. Science.

[11] Hurles M (2004). Gene duplication: the genomic trade in spare parts. PLoS Biol.

[12] Sandegren L, Andersson DI (2009). Bacterial gene amplification: implications for the evolution of antibiotic resistance. Nat Rev Microbiol.

[13] Gevers D, Vandepoele K, Simillon C, Van de Peer Y (2004). Gene duplication and biased functional retention of paralogs in bacterial genomes. Trends Microbiol.

[14] Koonin EV, Makarova KS, Aravind L (2001). Horizontal gene transfer in prokaryotes: quantification and classification. Annu Rev Microbiol.

[15] Lerat E, Daubin V, Ochman H, Moran NA (2005). Evolutionary origins of genomic repertoires in bacteria. PLoS Biol.

[16] Gogarten JP (1994). Which is the most conserved group of proteins? Homology-orthology, paralogy, xenology, and the fusion of independent lineages. J Mol Evol.

[17] Hooper SD, Berg OG (2003). Duplication is more common among laterally transferred genes than among indigenous genes. Genome Biol.

[18] Setubal JC, dos Santos P, Goldman BS, Ertesvåg H, Espin G, Rubio LM, Valla S, Almeida NF, Balasubramanian D, Cromes L, Curatti L, Du Z, Godsy E, Goodner B, Hellner-Burris K, Hernandez JA, Houmiel K, Imperial J, Kennedy C, Larson TJ, Latreille P, Ligon LS, Lu J, Maerk M, Miller NM, Norton S, O'Carroll IP, Paulsen I, Raulfs EC, Roemer R (2009). Genome sequence of *Azotobacter vinelandii*, an obligate aerobe specialized to support diverse anaerobic metabolic processes. J Bacteriol.

[19] Overbeek R, Begley T, Butler RM, Choudhuri JV, Chuang HY, Cohoon M, de Crécy-Lagard V, Diaz N, Disz T, Edwards R, Fonstein M, Frank ED, Gerdes S, Glass EM, Goesmann A, Hanson A, Iwata-Reuyl D, Jensen R, Jamshidi N, Krause L, Kubal M, Larsen N, Linke B, McHardy AC, Meyer F, Neuweger H, Olsen G, Olson R, Osterman A, Portnoy V (2005). The subsystems approach to genome annotation and its use in the project to annotate 1000 genomes. Nucleic Acids Res.

[20] Chen F, Mackey AJ, Stoeckert CJ, Roos DS (2006). OrthoMCL-DB: querying a comprehensive multi-species collection of ortholog groups. Nucleic Acids Res.

[21] Rocha EP (2006). Inference and analysis of the relative stability of bacterial chromosomes. Mol Biol Evol.

[22] Ballouz S, Francis AR, Lan R, Tanaka MM (2010). Conditions for the evolution of gene clusters in bacterial genomes. PLoS Comput Biol.

[23] Price MN, Arkin AP, Alm EJ (2006). The life-cycle of operons. PLoS Genet.

[24] Price MN, Huang KH, Arkin AP, Alm EJ (2005). Operon formation is driven by co-regulation and not by horizontal gene transfer. Genome Res.

[25] Noar JD, Bruno-Bárcena JM (2013). Complete genome sequences of *Azotobacter vinelandii* wild-type strain CA and tungsten-tolerant mutant strain CA6. Genome Announc.

[26] Bush JA, Wilson PW (1959). A non-gummy chromogenic strain of *Azotobacter vinelandii*. Nature.

[27] Wilson PW, Knight SG (1952). Experiments in bacterial physiology.

[28] Denef VJ, Banfield JF (2012). In situ evolutionary rate measurements show ecological success of recently emerged bacterial hybrids. Science.

[29] Konstantinidis KT, Tiedje JM (2004). Trends between gene content and genome size in prokaryotic species with larger genomes. Proc Natl Acad Sci U S A.

[30] Gu Z, Nicolae D, Lu HH, Li WH (2002). Rapid divergence in expression between duplicate genes inferred from microarray data. Trends Genet.

[31] Teichmann SA, Babu MM (2004). Gene regulatory network growth by duplication. Nat Genet.

[32] Starkenburg SR, Larimer FW, Stein LY, Klotz MG, Chain PS, Sayavedra-Soto LA, Poret-Peterson AT, Gentry ME, Arp DJ, Ward B, Bottomley PJ (2008). Complete genome sequence of *Nitrobacter hamburgensis* X14 and comparative genomic analysis of species within the genus *Nitrobacter*. Appl Environ Microbiol.

[33] Starkenburg SR, Arp DJ, Bottomley PJ (2008). D-Lactate metabolism and the obligate requirement for CO2 during growth on nitrite by the facultative lithoautotroph *Nitrobacter hamburgensis*. Microbiology.

[34] Han CS, Xie G, Challacombe JF, Altherr MR, Bhotika SS, Brown N, Bruce D, Campbell CS, Campbell ML, Chen J, Chertkov O, Cleland C, Dimitrijevic M, Doggett NA, Fawcett JJ, Glavina T, Goodwin LA, Green LD, Hill KK, Hitchcock P, Jackson PJ, Keim P, Kewalramani AR, Longmire J, Lucas S, Malfatti S, McMurry K, Meincke LJ, Misra M, Moseman BL (2006). Pathogenomic sequence analysis of *Bacillus cereus* and *Bacillus thuringiensis* isolates closely related to *Bacillus anthracis*. J Bacteriol.

[35] Erkel C, Kube M, Reinhardt R, Liesack W (2006). Genome of Rice Cluster I archaea - the key methane producers in the rice rhizosphere. Science.

[36] Galagan JE, Nusbaum C, Roy A, Endrizzi MG, Macdonald P, FitzHugh W, Calvo S, Engels R, Smirnov S, Atnoor D, Brown A, Allen N, Naylor J, Stange-Thomann N, DeArellano K, Johnson R, Linton L, McEwan P, McKernan K, Talamas J, Tirrell A, Ye W, Zimmer A, Barber RD, Cann I, Graham DE, Grahame DA, Guss AM, Hedderich R, Ingram-Smith C (2002). The genome of *M. acetivorans* reveals extensive metabolic and physiological diversity. Genome Res.

[37] Allen MA, Lauro FM, Williams TJ, Burg D, Siddiqui KS, De Francisci D, Chong KW, Pilak O, Chew HH, De Maere MZ, Ting L, Katrib M, Ng C, Sowers KR, Galperin MY, Anderson IJ, Ivanova N, Dalin E, Martinez M, Lapidus A, Hauser L, Land M, Thomas T, Cavicchioli R (2009). The genome sequence of the psychrophilic archaeon, *Methanococcoides burtonii*: the role of genome evolution in cold adaptation. ISME J.

[38] Deppenmeier U, Johann A, Hartsch T, Merkl R, Schmitz RA, Martinez-Arias R, Henne A, Wiezer A, Bäumer S, Jacobi C, Brüggemann H, Lienard T, Christmann A, Bömeke M, Steckel S, Bhattacharyya A, Lykidis A, Overbeek R, Klenk HP, Gunsalus RP, Fritz HJ, Gottschalk G (2002). The genome of *Methanosarcina mazei*: evidence for lateral gene transfer between bacteria and archaea. J Mol Microbiol Biotechnol.

[39] Sanchez-Perez G, Mira A, Nyiro G, Pasic L, Rodriguez-Valera F (2008). Adapting to environmental changes using specialized paralogs. Trends Genet.

[40] Bratlie MS, Johansen J, Sherman BT, Huang da W, Lempicki RA, Drabløs F (2010). Gene duplications in prokaryotes can be associated with environmental adaptation. BMC Genomics.

[41] Rost B (1999). Twilight zone of protein sequence alignments. Protein Eng.

[42] Blattner FR, Plunkett G, Bloch CA, Perna NT, Burland V, Riley M, Collado-Vides J, Glasner JD, Rode CK, Mayhew GF, Gregor J, Davis NW, Kirkpatrick HA, Goeden MA, Rose DJ, Mau B, Shao Y (1997). The complete genome sequence of *Escherichia coli* K-12. Science.

[43] Toft C, Andersson SG (2010). Evolutionary microbial genomics: insights into bacterial host adaptation. Nat Rev Genet.

[44] Nakayama K, Yamashita A, Kurokawa K, Morimoto T, Ogawa M, Fukuhara M, Urakami H, Ohnishi M, Uchiyama I, Ogura Y, Ooka T, Oshima K, Tamura A, Hattori M, Hayashi T (2008). The whole-genome sequencing of the obligate intracellular bacterium *Orientia tsutsugamushi* revealed massive gene amplification during reductive genome evolution. DNA Res.

[45] Cho NH, Kim HR, Lee JH, Kim SY, Kim J, Cha S, Kim SY, Darby AC, Fuxelius HH, Yin J, Kim JH, Kim J, Lee SJ, Koh YS, Jang WJ, Park KH, Andersson SG, Choi MS, Kim IS (2007). The *Orientia tsutsugamushi* genome reveals massive proliferation of conjugative type IV secretion system and host-cell interaction genes. Proc Natl Acad Sci U S A.

[46] Bai X, Zhang J, Ewing A, Miller SA, Jancso Radek A, Shevchenko DV, Tsukerman K, Walunas T, Lapidus A, Campbell JW, Hogenhout SA (2006). Living with genome instability: the adaptation of phytoplasmas to diverse environments of their insect and plant hosts. J Bacteriol.

[47] Tran-Nguyen LT, Kube M, Schneider B, Reinhardt R, Gibb KS (2008). Comparative genome analysis of “*Candidatus* Phytoplasma australiense” (subgroup tuf-Australia I; rp-A) and “*Ca*. Phytoplasma asteris” Strains OY-M and AY-WB. J Bacteriol.

[48] Salzberg SL, Sommer DD, Schatz MC, Phillippy AM, Rabinowicz PD, Tsuge S, Furutani A, Ochiai H, Delcher AL, Kelley D, Madupu R, Puiu D, Radune D, Shumway M, Trapnell C, Aparna G, Jha G, Pandey A, Patil PB, Ishihara H, Meyer DF, Szurek B, Verdier V, Koebnik R, Dow JM, Ryan RP, Hirata H, Tsuyumu S, Won Lee S, Seo YS (2008). Genome sequence and rapid evolution of the rice pathogen *Xanthomonas oryzae* pv. oryzae PXO99A. BMC Genomics.

[49] Yang F, Yang J, Zhang X, Chen L, Jiang Y, Yan Y, Tang X, Wang J, Xiong Z, Dong J, Xue Y, Zhu Y, Xu X, Sun L, Chen S, Nie H, Peng J, Xu J, Wang Y, Yuan Z, Wen Y, Yao Z, Shen Y, Qiang B, Hou Y, Yu J, Jin Q (2005). Genome dynamics and diversity of *Shigella* species, the etiologic agents of bacillary dysentery. Nucleic Acids Res.

[50] Casjens S, Palmer N, van Vugt R, Huang WM, Stevenson B, Rosa P, Lathigra R, Sutton G, Peterson J, Dodson RJ, Haft D, Hickey E, Gwinn M, White O, Fraser CM (2000). A bacterial genome in flux: the twelve linear and nine circular extrachromosomal DNAs in an infectious isolate of the Lyme disease spirochete *Borrelia burgdorferi*. Mol Microbiol.

[51] Fraser CM, Casjens S, Huang WM, Sutton GG, Clayton R, Lathigra R, White O, Ketchum KA, Dodson R, Hickey EK, Gwinn M, Dougherty B, Tomb JF, Fleischmann RD, Richardson D, Peterson J, Kerlavage AR, Quackenbush J, Salzberg S, Hanson M, van Vugt R, Palmer N, Adams MD, Gocayne J, Weidman J, Utterback T, Watthey L, McDonald L, Artiach P, Bowman C (1997). Genomic sequence of a Lyme disease spirochaete, *Borrelia burgdorferi*. Nature.

[52] Westberg J, Persson A, Holmberg A, Goesmann A, Lundeberg J, Johansson KE, Pettersson B, Uhlen M (2004). The genome sequence of *Mycoplasma mycoides* subsp. *mycoides* SC type strain PG1T, the causative agent of contagious bovine pleuropneumonia (CBPP). Genome Res.

[53] Klasson L, Westberg J, Sapountzis P, Näslund K, Lutnaes Y, Darby AC, Veneti Z, Chen L, Braig HR, Garrett R, Bourtzis K, Andersson SG (2009). The mosaic genome structure of the *Wolbachia w*Ri strain infecting *Drosophila simulans*. Proc Natl Acad Sci U S A.

[54] Engel P, Dehio C (2009). Genomics of host-restricted pathogens of the genus *Bartonella*. Genome Dyn.

[55] Berglund EC, Frank AC, Calteau A, Vinnere Pettersson O, Granberg F, Eriksson AS, Naslund K, Holmberg M, Lindroos H, Andersson SG (2009). Run-off replication of host-adaptability genes is associated with gene transfer agents in the genome of mouse-infecting *Bartonella grahamii*. PLoS Genet.

[56] Lee BM, Park YJ, Park DS, Kang HW, Kim JG, Song ES, Park IC, Yoon UH, Hahn JH, Koo BS, Lee GB, Kim H, Park HS, Yoon KO, Kim JH, Jung CH, Koh NH, Seo JS, Go SJ (2005). The genome sequence of *Xanthomonas oryzae* pathovar oryzae KACC10331, the bacterial blight pathogen of rice. Nucleic Acids Res.

[57] Ochiai H, Inoue Y, Takeya M, Sasaki A, Kaku H (2005). Genome sequence of Xanthomonas oryzae pv. oryzae suggests contribution of large numbers of effector genes and insertion sequences to its race diversity. Jpn Agric Res Q.

[58] Jin Q, Yuan Z, Xu J, Wang Y, Shen Y, Lu W, Wang J, Liu H, Yang J, Yang F, Zhang X, Zhang J, Yang G, Wu H, Qu D, Dong J, Sun L, Xue Y, Zhao A, Gao Y, Zhu J, Kan B, Ding K, Chen S, Cheng H, Yao Z, He B, Chen R, Ma D, Qiang B (2002). Genome sequence of *Shigella flexneri* 2a: insights into pathogenicity through comparison with genomes of *Escherichia coli* K12 and O157. Nucleic Acids Res.

[59] Wei J, Goldberg MB, Burland V, Venkatesan MM, Deng W, Fournier G, Mayhew GF, Plunkett G, Rose DJ, Darling A, Mau B, Perna NT, Payne SM, Runyen-Janecky LJ, Zhou S, Schwartz DC, Blattner FR (2003). Complete genome sequence and comparative genomics of *Shigella flexneri* serotype 2a strain 2457 T. Infect Immun.

[60] Kaneko T, Nakajima N, Okamoto S, Suzuki I, Tanabe Y, Tamaoki M, Nakamura Y, Kasai F, Watanabe A, Kawashima K, Kishida Y, Ono A, Shimizu Y, Takahashi C, Minami C, Fujishiro T, Kohara M, Katoh M, Nakazaki N, Nakayama S, Yamada M, Tabata S, Watanabe MM (2007). Complete genomic structure of the bloom-forming toxic cyanobacterium *Microcystis aeruginosa* NIES-843. DNA Res.

[61] Klasson L, Walker T, Sebaihia M, Sanders MJ, Quail MA, Lord A, Sanders S, Earl J, O'Neill SL, Thomson N, Sinkins SP, Parkhill J (2008). Genome evolution of *Wolbachia* strain wPip from the Culex pipiens group. Mol Biol Evol.

[62] Degnan PH, Yu Y, Sisneros N, Wing RA, Moran NA (2009). *Hamiltonella defensa*, genome evolution of protective bacterial endosymbiont from pathogenic ancestors. Proc Natl Acad Sci U S A.

[63] Lescot M, Audic S, Robert C, Nguyen TT, Blanc G, Cutler SJ, Wincker P, Couloux A, Claverie JM, Raoult D, Drancourt M (2008). The genome of *Borrelia recurrentis*, the agent of deadly louse-borne relapsing fever, is a degraded subset of tick-borne *Borrelia duttonii*. PLoS Genet.

[64] She Q, Singh RK, Confalonieri F, Zivanovic Y, Allard G, Awayez MJ, Chan-Weiher CC, Clausen IG, Curtis BA, De Moors A, Erauso G, Fletcher C, Gordon PM, Heikamp-de Jong I, Jeffries AC, Kozera CJ, Medina N, Peng X, Thi-Ngoc HP, Redder P, Schenk ME, Theriault C, Tolstrup N, Charlebois RL, Doolittle WF, Duguet M, Gaasterland T, Garrett RA, Ragan MA, Sensen CW (2001). The complete genome of the crenarchaeon *Sulfolobus solfataricus* P2. Proc Natl Acad Sci U S A.

[65] Hjerde E, Lorentzen MS, Holden MT, Seeger K, Paulsen S, Bason N, Churcher C, Harris D, Norbertczak H, Quail MA, Sanders S, Thurston S, Parkhill J, Willassen NP, Thomson N (2008). The genome sequence of the fish pathogen *Aliivibrio salmonicida* strain LFI1238 shows extensive evidence of gene decay. BMC Genomics.

[66] Vallenet D, Nordmann P, Barbe V, Poirel L, Mangenot S, Bataille E, Dossat C, Gas S, Kreimeyer A, Lenoble P, Oztas S, Poulain J, Segurens B, Robert C, Abergel C, Claverie JM, Raoult D, Médigue C, Weissenbach J, Cruveiller S (2008). Comparative analysis of *Acinetobacters*: three genomes for three lifestyles. PLoS One.

[67] Swingley WD, Chen M, Cheung PC, Conrad AL, Dejesa LC, Hao J, Honchak BM, Karbach LE, Kurdoglu A, Lahiri S, Mastrian SD, Miyashita H, Page L, Ramakrishna P, Satoh S, Sattley WM, Shimada Y, Taylor HL, Tomo T, Tsuchiya T, Wang ZT, Raymond J, Mimuro M, Blankenship RE, Touchman JW (2008). Niche adaptation and genome expansion in the chlorophyll d-producing cyanobacterium *Acaryochloris marina*. Proc Natl Acad Sci U S A.

[68] Brugger K, Redder P, She Q, Confalonieri F, Zivanovic Y, Garrett RA (2002). Mobile elements in archaeal genomes. FEMS Microbiol Lett.

[69] Pfeiffer F, Schuster SC, Broicher A, Falb M, Palm P, Rodewald K, Ruepp A, Soppa J, Tittor J, Oesterhelt D (2008). Evolution in the laboratory: the genome of *Halobacterium salinarum* strain R1 compared to that of strain NRC-1. Genomics.

[70] Ogata H, Renesto P, Audic S, Robert C, Blanc G, Fournier PE, Parinello H, Claverie JM, Raoult D (2005). The genome sequence of *Rickettsia felis* identifies the first putative conjugative plasmid in an obligate intracellular parasite. PLoS Biol.

[71] *The SEED Servers*. [http://blog.theseed.org/servers/]

[72] Larkin MA, Blackshields G, Brown NP, Chenna R, McGettigan PA, McWilliam H, Valentin F, Wallace IM, Wilm A, Lopez R, Thompson JD, Gibson TJ, Higgins DG (2007). Clustal W and Clustal X version 2.0. Bioinformatics.

[73] Felsenstein J (1989). PHYLIP - Phylogeny Inference Package (Version 3.2). Cladistics.

[74] *Python Programming Language - Official Website*. [http://www.python.org/]

[75] FTP Server: *Genomes; bacteria*. [ftp://ftp.ncbi.nih.gov/genomes/Bacteria/]

[76] Altschul SF, Madden TL, Schaffer AA, Zhang J, Zhang Z, Miller W, Lipman DJ (1997). Gapped BLAST and PSI-BLAST: a new generation of protein database search programs. Nucleic Acids Res.

