# Supplementary material for: Safety in numbers: multiple occurrences of highly similar homologs among Azotobacter vinelandii carbohydrate metabolism proteins probably confer adaptive benefits
Source: BMC Genomics. 2014 Mar 14;15(1):192. doi: 10.1186/1471-2164-15-192 (PMC4022178; doi:10.1186/1471-2164-15-192)

# Additional file 1

**Supplementary Table S1 - Genomes where highly similar synologs constitute ≥10% of the total number of protein-coding genes**

The synolog fraction describes the ratio of the total number of synologs relative to the total number of protein-coding sequences. Synolog fractions for synologs with at least 90% identity at amino acid level are shown.

| Genome | Genome size (Mb) | Synolog fraction of total proteins |
|---|---|---|
| *Orientia tsutsugamushi* str. Ikeda | 2.0 | 52% |
| *Orientia tsutsugamushi* str. Boryong | 2.1 | 33% |
| *Candidatus* Phytoplasma australiense | 0.9 | 26% |
| *Bartonella tribocorum* CIP 105476 | 2.6 | 24% |
| *Xanthomonas oryzae* pv. *oryzae* PXO99A | 5.2 | 24% |
| Onion yellows *phytoplasma* OY-M | 0.9 | 23% |
| *Shigella dysenteriae* M131649 | 4.6 | 23% |
| *Mycoplasma mycoides* subsp. *mycoides* SC str. PG1 | 1.2 | 20% |
| *Borrelia burgdorferi* B31 | 1.5 | 19% |
| *Shigella boydii* CDC 3083-94 | 4.9 | 18% |
| *Microcystis aeruginosa* NIES-843 | 5.8 | 18% |
| *Wolbachia* sp. wRi | 1.4 | 17% |
| *Xanthomonas oryzae* pv. *oryzae* KACC 10331 | 4.9 | 17% |
| *Xanthomonas oryzae* pv. *oryzae* MAFF 311018 | 4.9 | 17% |
| *Shigella boydii* Sb227 | 4.6 | 17% |
| *Neisseria meningitidis* MC58 | 2.3 | 16% |
| *Shigella flexneri* 2a | 4.8 | 16% |
| *Wolbachia* endosymbiont of *Culex quinquefasciatus* Pel strain wPip | 1.5 | 16% |
| Aster yellows witches'-broom *phytoplasma* AYWB | 5.1 | 16% |
| *Vibrio harveyi* ATCC BAA-1116 | 6.1 | 15% |
| *Magnetococcus marinus* MC-1 | 4.7 | 15% |
| *Shigella sonnei* Ss046 | 5.0 | 14% |
| Candidatus *Phytoplasma mali* | 0.6 | 14% |
| *Candidatus Hamiltonella defensa* 5AT (*Acyrthosiphon pisum*) | 2.2 | 13% |
| *Shigella flexneri* 2a str. 2457T | 4.6 | 13% |
| *Shigella flexneri* 5 str. 8401 | 4.6 | 13% |
| *Sulfolobus solfataricus* P2 | 3.0 | 13% |
| *Aliivibrio salmonicida* LFI1238 | 4.6 | 13% |
| *Borrelia duttonii* Ly | 1.6 | 12% |
| *Acinetobacter baumannii* SDF | 3.5 | 12% |
| *Acaryochloris marina* MBIC11017 | 8.4 | 12% |
| *Escherichia coli* O157:H7 str. EDL933 | 5.6 | 12% |
| *Halobacterium* sp. NRC-1 | 2.6 | 12% |
| *Yersinia pestis* biovar Mediaevalis | 4.8 | 12% |
| *Escherichia coli* O157:H7 str. EC4115 | 5.7 | 12% |
| *Methylobacterium nodulans* ORS 2060 | 8.9 | 11% |
| *Methylobacillus flagellatus* KT | 3.0 | 11% |
| *Halobacterium salinarum* R1 | 2.7 | 10% |
| *Rickettsia felis* URRWXCa12 | 1.6 | 10% |
| *Shewanella baltica* OS155 | 5.3 | 10% |

**Supplementary Figure S1 - Distribution of number of proteins, synologs and synolog groups, and synolog fractions, independent of functional categories**

Distribution of **a)** total number of protein-coding sequences (skewness = 0.57), **b)** total number of synolog groups (skewness = 3.25) and **c)** synolog fractions (skewness = 4.62) in a data set consisting of 897 bacterial and archaeal genomes. Synologs were identified at a threshold of 90% protein sequence identity. This shows that the majority of the analysed genomes have a low content of highly similar synologs. The synolog fraction describes the ratio of the total number of synologs relative to the total number of proteins for a given proteome. Whiskers represent the 10th and 90th percentile and dots represent outliers.

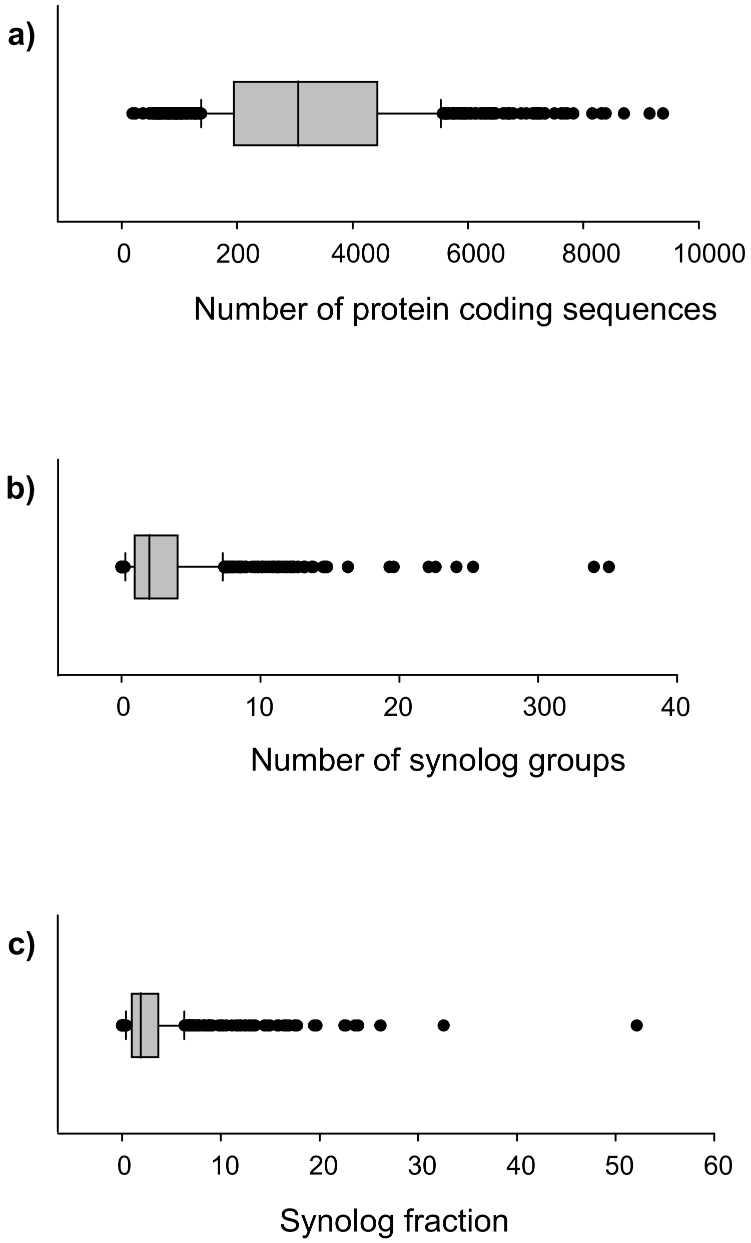

**Supplementary Figure S2 - Distribution of difference between best and worst match for comparisons of synolog pairs retrieved from the SEED database**

Calculation of sequence identity from pairwise alignments is sensitive to differences in sequence length, caused e.g. by a gene fusion or a severely truncated gene product. The figure shows the distribution of the difference between the best and worst match (%), corresponding to normalisation against the shortest or longest sequence in the alignment, respectively. This shows that for sequence comparisons of synolog pairs retrieved from the SEED database (see Methods) the difference is minimal for the large majority of the alignments.

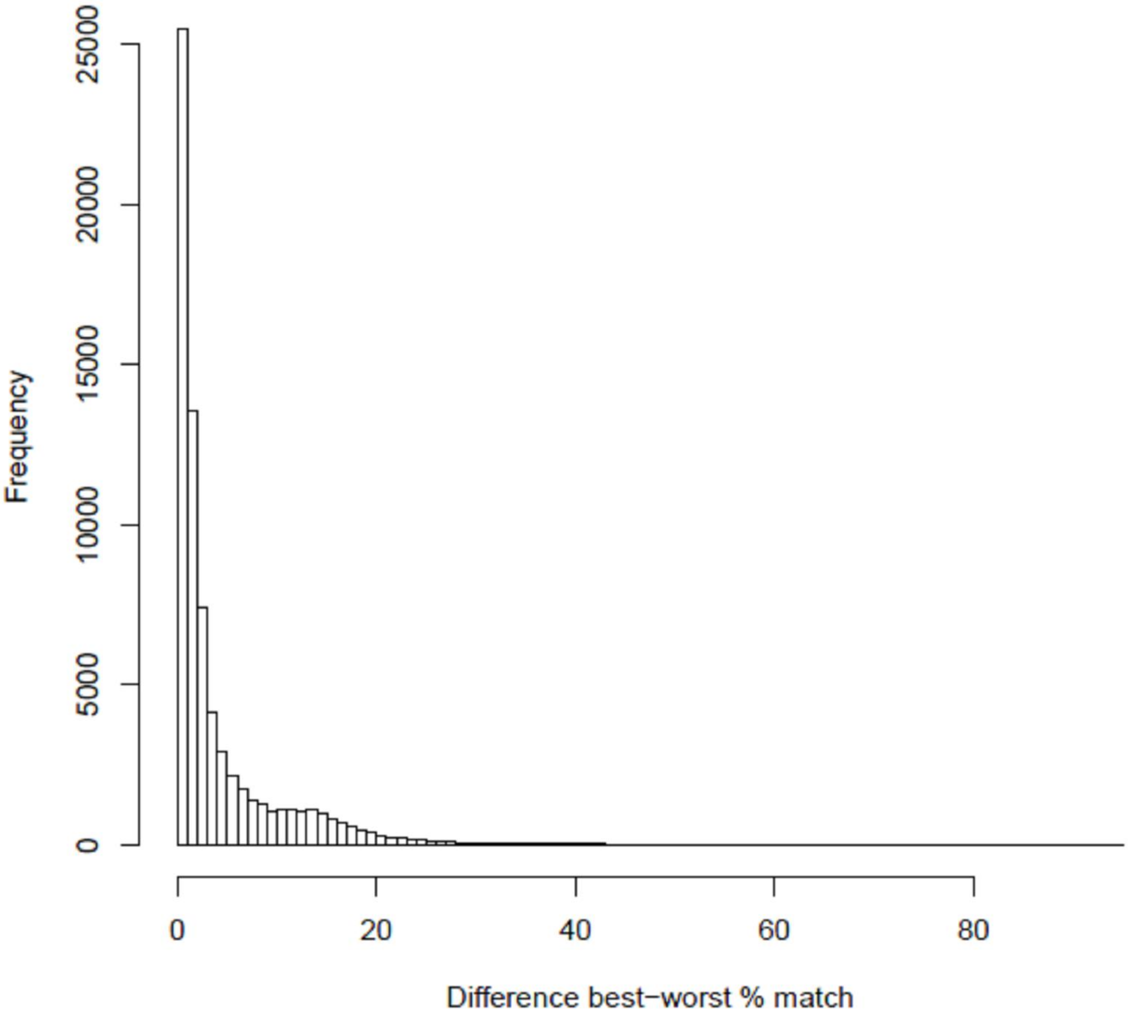

Supplement: Supplementary file 1 — Additional file 1: Table S1: Genomes where highly similar synologs constitute ≥10% of the total number of protein-coding genes. Figure S1. Distribution of number of proteins, synologs and synolog groups, and synolog fractions, independent of functional categories. Figure S2. Distribution of difference between best and worst match for comparisons of synolog pairs retrieved from the SEED database. (PDF 173 KB) [file 12864_2013_7030_MOESM1_ESM.pdf]
